# Gray matter microstructural alterations and their correlation with systemic biomarkers in hepatic encephalopathy: a NODDI study using gray-matter based spatial statistics

**DOI:** 10.3389/fneur.2026.1783288

**Published:** 2026-03-02

**Authors:** Fengli Xie, Xiaohui Wang, Huina Zhang, Juan Wang, Shaofeng Wang, Peng Cheng, Jiangong Zhou, Haohui Zhan

**Affiliations:** Medical Imaging Center, The Second Affiliated Hospital of Henan University of Science and Technology, Luoyang, China

**Keywords:** biomarkers, globus pallidus, gray-matter based spatial statistics, hepatic encephalopathy, neurite orientation dispersion and density imaging

## Abstract

**Background:**

Hepatic encephalopathy (HE) involves complex neurobiological changes that are often difficult to quantify using conventional MRI. This study aims to utilize Neurite Orientation Dispersion and Density Imaging (NODDI) combined with Gray-matter Based Spatial Statistics (GBSS) to characterize microstructural alterations in patients with HE and explore their relationship with clinical biochemical markers, specifically within the globus pallidus (GP).

**Methods:**

Thirty-three patients with HE and 31 healthy controls underwent 3 T MRI including a multi-shell diffusion protocol for NODDI. GBSS was performed to assess differences in the Neurite Density Index (NDI) and Orientation Dispersion Index (ODI). Pearson correlation analyzed relationships between GP NODDI parameters and blood biochemical indices.

**Results:**

HE patients exhibited significantly decreased NDI across widespread cortical and sub-cortical regions (frontal, parietal, temporal, cingulate, insula, thalamus) and increased ODI in the posterior cerebellum/vermis. Exploratory ROI analysis of the globus pallidus (GP)—a region known for manganese deposition but showing no significant group-level differences in this study- revealed that, the NDI of the right GP showed positive correlations with indirect bilirubin and prothrombin international normalized ratio (all uncorrected *p* < 0.05), while the ODI of the left GP positively correlated with hemoglobin concentration (uncorrected *p* = 0.046).

**Conclusion:**

NODDI reveals extensive microstructural alterations consistent with reduced neurite density index and cerebellar disorganization in HE. The dissociated correlation patterns of GP NDI and ODI with distinct blood markers may be compatible with a hypothetical “double-hit” pathophysiological model: toxic metabolite accumulation may drive cellular swelling (increased NDI), while systemic factors like anemia may reduce structural complexity (decreased ODI). However, these exploratory associations do not allow causal inference. These findings highlight NODDI could be a useful tool for monitoring the progression and metabolic impact of HE.

## 1 Introduction

Hepatic encephalopathy (HE) is a common neurological complication in patients with cirrhosis, manifesting as a spectrum of neuropsychiatric symptoms ranging from subtle cognitive impairment to coma (1).

With the aging of the global population, the differential diagnosis between HE and neurodegenerative disorders has become increasingly challenging due to overlapping clinical presentations (2). Traditional diagnostic approaches primarily rely on clinical assessment and blood biochemical markers such as ammonia levels, but these methods are limited by subjectivity and insufficient sensitivity (3).

Among various neuroimaging biomarkers, the globus pallidus (GP) has garnered significant attention in HE research. Magnetic resonance imaging (MRI) studies have demonstrated that 70–90% of HE patients exhibit bilateral symmetric hyperintensity in the GP on T1-weighted images (4). The relationship between this pallidal T1 hyperintensity and plasma ammonia levels remains a subject of debate. While some studies have identified significant associations between pallidal signal intensity and ammonia concentration (5, 6), others have failed to find such a correlation (7). This suggests that pallidal T1 hyperintensity is primarily linked to manganese-related neurotoxicity but may also involve additional mechanisms that are partly distinct from hyperammonemia (8).

While conventional structural MRI captures macroscopic changes, it offers limited sensitivity to microstructural alterations. Neurite Orientation Dispersion and Density Imaging (NODDI) is an advanced diffusion MRI technique that enables non-invasive quantification of brain tissue microstructure through parameters including the Neurite Density Index (NDI) and Orientation Dispersion Index (ODI) (9). These metrics provide more refined biological markers for investigating HE-related cerebral microstructural abnormalities. Recent studies have begun applying NODDI to explore microstructural changes in HE, though most have focused primarily on white matter alterations (10).

Gray-matter Based Spatial Statistics (GBSS) is a voxel-based analysis method specifically designed to investigate spatial patterns of gray matter microstructure (11). A recent study combining NODDI with GBSS successfully identified cortical microstructural abnormalities in patients with minimal hepatic encephalopathy (12). However, this research did not specifically focus on the GP—a region known to be particularly vulnerable in HE—nor did it systematically examine the correlation between cerebral microstructural parameters and blood biochemical indices.

Given these research gaps, the study aims to: (i) assess brain microstructural alterations in patients with HE using NODDI combined with GBSS methodology; (ii) analyze correlations between the NODDI parameters of the GP in the HE group and blood biochemical indices to provide novel imaging evidence for understanding the pathophysiological mechanisms underlying HE.

## 2 Materials and methods

### 2.1 Participants

The study was approved by the local Ethics Committee (no. L2023005) and all participants provided written informed consent (version V1.0; dated August 9, 2023).

Thirty-three patients clinically diagnosed with decompensated cirrhosis between December 2023 and June 2025 were enrolled in this study, and 31 healthy volunteers were included as the health control (HC) group. The inclusion criteria for the HE group were as follows: (i) Clinical diagnosis of decompensated cirrhosis; (ii) Imaging evidence of cirrhosis, portal hypertension, and ascites as shown by imaging examinations. The inclusion criteria for the control group were: (i) routine health examination results indicating good health with no known significant systemic organ diseases; (ii) laboratory tests (including complete blood count, liver and kidney function, coagulation profile, and other major biochemical indices) within normal ranges. The exclusion criteria for both groups included: (i) contraindications to MRI (e.g., presence of cardiac pacemakers, cochlear implants, or metallic implants) that prevent the completion of scanning; (ii) poor image quality rendering the data unsuitable for subsequent analysis; (iii) incomplete data, with failure to complete all study procedures (such as MRI scanning and blood biochemical tests); (iv) long-term abuse of substances or medications that may affect the central nervous system (e.g., antipsychotics, sedatives).

### 2.2 MRI data acquisition

MRI examinations were performed on a 3.0-T scanner (SIGNA Architect 3.0 T, GE Healthcare, US) with a combined head and neck coil. Diffusion-weighted magnetic resonance imaging (dMRI) was performed using a spin-echo planar imaging sequence with the following parameters: repetition time (TR) = 4,286 ms; echo time (TE) = 113.5 ms; field of view (FOV) = 240 × 240 mm^2^; matrix = 128 × 128; slice thickness = 3 mm; non-diffusion weighted images (b = 0 s/mm^2^) as well as 30 noncollinear directions with multiple b values (b = 1,000, 2,500 s/mm^2^). T1-weighted structural images were acquired using a 3D Gradient-Echo (GRE) BRAin VOlume (known as BRAVO) sequences with the following parameters: TR/TE = 6.3/2.4 ms; FOV = 256 × 256 × 160 mm^3^; voxel size = 0.5 × 0.5 × 0.5 mm^3^.

### 2.3 Diffusion-weighted data processing

First, the MRIcroGL software^1^ was used to convert all raw data in DICOM format into NIFTI format, and further anonymize patient data. Then, the dMRI were processed using the EDDY and TOPUP tools from the FMRIB Software Library (FSL) to perform eddy current correction, geometric distortion correction, and head motion correction. Following these preprocessing steps, the diffusion tensor model was applied to the corrected data, and fractional anisotropy (FA) maps were generated using the weighted linear least squares method implemented in the DIPY library. Subsequently, the Accelerated Microstructure Imaging via Convex Optimization (AMICO) approach was utilized to compute parameters associated with the NODDI model. This analysis yielded key metrics, including the Neurite Density Index (NDI), Orientation Dispersion Index (ODI), and free water fraction (FWF).

### 2.4 Post-processing of GBSS

**Figure 1:**
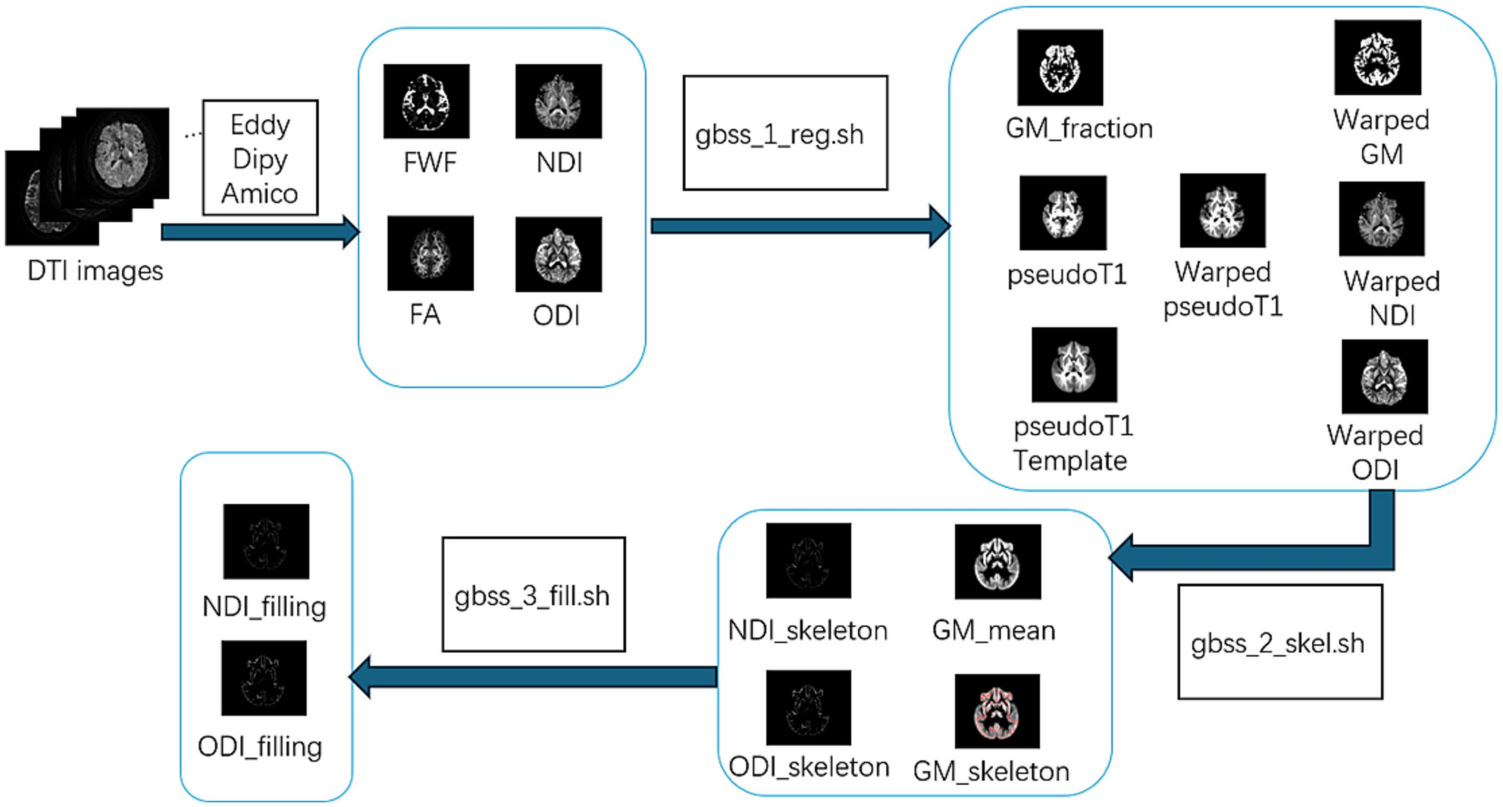
Processing pipeline of the Gray Matter-Boundary Structural Skeleton (GBSS). FWF, free water fraction; NDI, neurite density index; ODI, orientation dispersion index; FA, fractional ani-sotropy; GM, gray matter.

GBSS was performed to analyze the microstructural changes of gray matter using scripts available online.^2^ The specific steps are illustrated in Figure 1. Firstly, gray matter fraction maps were derived in the native diffusion space by subtracting FWF and white matter fractions from unity in each voxel. The FWF were obtained from NODDI, while the white matter fractions were estimated using two-tissue class segmentation of FA images with Atropos. A study-specific pseudo-T1 template was created through iterative group averaging of subject-level pseudo-T1 images, which were derived from the weighted fusion of white matter and gray matter fraction maps. The preprocessed outputs were then input into the gbss_1_reg.sh script for affine registration, followed by non-linear spatial registration to this custom template. This process generated a suite of registered derivatives, including gray matter fraction maps, warped pseudo-T1 images, and warped gray matter, NDI, and ODI maps. Subsequently, the gbss_2_skel.sh script was employed to process these registered maps and extract skeletonized features, namely NDI_skeleton, ODI_skeleton, and GM_skeleton, along with a mean gray matter map. Skeletonization was constrained by a thresholded average gray matter fraction mask to preserve region-specific structural boundaries. Finally, the skeletonized products were refined using the gbss_3_fill.sh script, which applied a voxel-wise nearest-neighbor smoothing algorithm to produce fully filled, spatially complete NDI_filling and ODI_filling metrics for subsequent downstream GBSS statistical analysis.

### 2.5 Blood biochemical examination

All patients underwent Blood Biochemical Examination, including liver synthetic function (Albumin, International Normalized Ratio [INR]), cholestasis (Indirect Bilirubin), and other relevant factors (D-dimer, Hemoglobin concentration).

### 2.6 Statistical analysis

Randomization and threshold-free cluster enhancement (TFCE) were performed within the FSL to explore differences in NDI_filling and ODI_filling metrics between the HE and HC group. A nonparametric permutation test with 1,000 iterations was conducted. Family-wise error (FWE) correction was performed to address multiple comparisons, with a significant threshold set at PFWE<0.05.

The relationship between NODDI parameters of the GP (NDI and ODI) and blood biochemical indices (6 markers) in the HE group was investigated using Pearson correlation analysis. A total of 12 correlation tests were performed. Given the exploratory nature of these analyses, no formal correction for multiple comparisons was applied, and a *p*-value < 0.05 was considered to indicate a potentially significant association requiring further validation.

## 3 Results

The demographic information is shown in Table 1.

**Table 1:** Demographic information the hepatic encephalopathy (HE) and health control (HC) groups.

Parameters	HE (*n* = 33)	HC (*n* = 31)	*p* value
Female/Male	9/24	13/18	0.326
Age (Year)	63.7 ± 11.4	59.3 ± 6.2	0.078
Hematological indices			
Platelet count (10^9^/L)	77.00 (60.50, 140.50)	-	-
White blood cell count (10^9^/L)	4.68 (3.81, 7.12)	-	-
Hemoglobin (g/L)	105.56 ± 24.70	-	-
Liver function			
ALT (U/L)	33.30 (22.60, 63.80)	-	-
AST (U/L)	58.10 (40.00, 92.80)	-	-
Total bilirubin (μmol/L)	26.40 (22.22, 70.30)	-	-
Direct bilirubin (μmol/L)	14.06 (6.63, 33.52)	-	-
Indirect bilirubin (μmol/L)	13.82 (10.30, 23.00)	-	-
ALP (U/L)	118.10 (80.00, 146.70)	-	-
Albumin (g/L)	35.40 (30.60, 40.00)	-	-
Coagulation profile			
PT (s)	13.80 (12.50, 15.60)	-	-
TT (s)	19.60 (18.15, 22.40)	-	-
APTT (s)	32.50 (29.20, 38.00)	-	-
Fibrinogen (g/L)	2.40 (1.54, 2.90)	-	-
D-dimer (μg/L)	3320.00 (1540.00, 5510.00)	-	-
INR	1.17 (1.05, 1.33)	-	-

Data are presented as mean ± SD for normally distributed variables and median (interquartile range, IQR) for non-normally distributed variables. ALP, alkaline phosphatase; ALT, alanine aminotransferase; APTT, activated partial thromboplastin time; AST, aspartate aminotransferase; INR, international normalized ratio; NDI, neurite density index; ODI, orientation dispersion index; PT, prothrombin time; TT, thrombin time.

### 3.1 Significant group differences between the HE and HC group in terms of NDI

**Figure 2:**
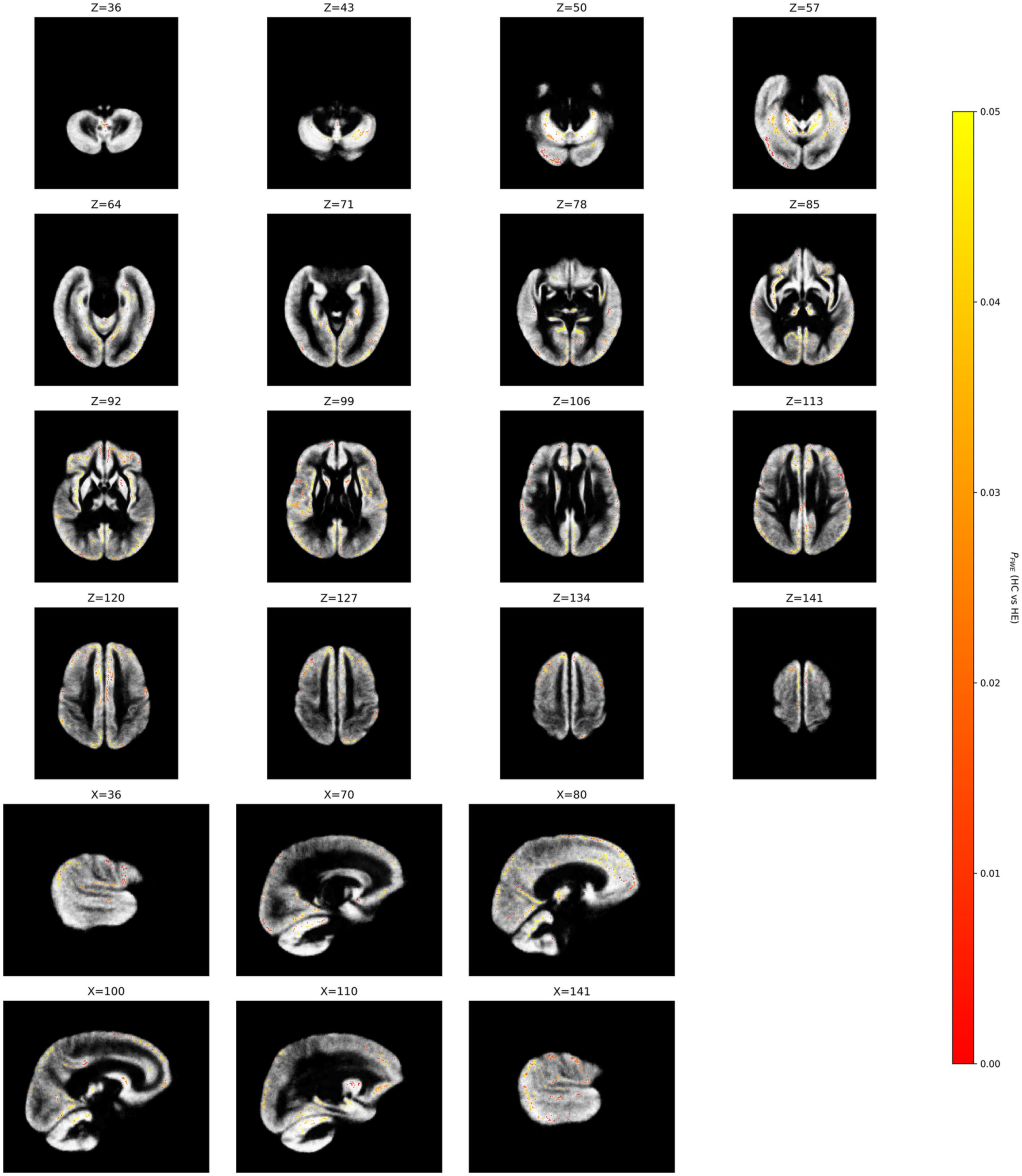
Between-group differences in neurite density index (NDI). Yellow clusters reflect GM decreases in the Hepatic encephalopathy (HE) group. The color bar represents the *p* value. Significance is indicated by *p* < 0.05, FWE corrected.

In patients with HE, a significantly decreased NDI was found in some regions of the gray matter, primarily the right frontal cortex (including the right medial superior frontal gyrus, right anterior cingulate gyrus, right superior frontal gyrus, right supplementary motor area, and right inferior frontal gyrus), bilateral parietal cortex (including the bilateral precuneus, bilateral superior parietal gyri, right angular gyrus, and right inferior parietal gyrus), left occipital regions (left cuneus, left calcarine gyrus, left fusiform gyrus, and left middle occipital gyrus), left temporal cortex (including the left middle temporal gyrus and left temporal pole), right insula, bilateral middle cingulate gyri, bilateral posterior cingulate gyrus, right hippocampus, bilateral parahippocampal gyri, left thalamus, right putamen and bilateral cerebellum. In addition to these primary clusters, several other regions showed isolated voxels or very small clusters of alteration (Figure 2; Table 2).

**Table 2:** Brain regions with significant differences in neurite density index (NDI) between the hepatic encephalopathy (HE) and health control (HC) groups.

Cluster	Voxels	Coordinates			P_peak value_	Cohen’s d	AAL3 atlas
		X	Y	Z			
1	969	−9	274	51	0.008	1.36	Frontal_Sup_Medial_R (Voxels: 511, AAL ID: 24); Cingulum_Mid_R (Voxels: 225, AAL ID: 34); Cingulum_Ant_R (Voxels: 139, AAL ID: 32); Frontal_Sup_R (Voxels: 54, AAL ID: 4); Supp_Motor_Area_R (Voxels: 22, AAL ID: 20); Cingulum_Mid_L (Voxels: 10, AAL ID: 33); Frontal_Mid_R (Voxels: 2, AAL ID: 8); Frontal_Sup_Medial_L (Voxels: 2, AAL ID: 23)
2	789	−35	249	19	0.005	1.37	Insula_R (Voxels: 216, AAL ID: 30); Putamen_R (Voxels: 80, AAL ID: 74); Frontal_Inf_Orb_R (Voxels: 27, AAL ID: 16)
3	737	−1	204	−18	0.01	1.60	Cerebelum_4_5_L (Voxels: 174, AAL ID: 97); Cerebelum_4_5_R (Voxels: 91, AAL ID: 98); Cerebelum_6_L (Voxels: 61, AAL ID: 99); Vermis_8 (Voxels: 57, AAL ID: 114); Vermis_4_5 (Voxels: 54, AAL ID: 111); Fusiform_L (Voxels: 38, AAL ID: 55); Cerebelum_8_L (Voxels: 23, AAL ID: 103); Vermis_6 (Voxels: 22, AAL ID: 112); Cerebelum_8_R (Voxels: 19, AAL ID: 104); Cerebelum_6_R (Voxels: 12, AAL ID: 100); Vermis_9 (Voxels: 12, AAL ID: 115); Cerebelum_Crus2_R (Voxels: 2, AAL ID: 94); Vermis_7 (Voxels: 1, AAL ID: 113); ParaHippocampal_L (Voxels: 1, AAL ID: 39)
4	398	−6	181	28	0.015	1.61	Cuneus_L (Voxels: 135, AAL ID: 45); Precuneus_L (Voxels: 119, AAL ID: 67); Cingulum_Post_L (Voxels: 28, AAL ID: 35); Calcarine_L (Voxels: 19, AAL ID: 43); Precuneus_R (Voxels: 18, AAL ID: 68); Cingulum_Post_R (Voxels: 4, AAL ID: 36); Calcarine_R (Voxels: 2, AAL ID: 44)
5	252	40	173	26	0.015	1.35	Temporal_Mid_L (Voxels: 26, AAL ID: 85); Occipital_Mid_L (Voxels: 4, AAL ID: 51)
6	210	−9	176	45	0.024	1.30	Precuneus_L (Voxels: 67, AAL ID: 67); Precuneus_R (Voxels: 58, AAL ID: 68); Parietal_Sup_R (Voxels: 27, AAL ID: 60)
7	116	22	195	−26	0.022	1.39	Cerebelum_Crus1_L (Voxels: 48, AAL ID: 91); Cerebelum_6_L (Voxels: 41, AAL ID: 99)
8	112	10	171	51	0.02	1.54	Parietal_Sup_L (Voxels: 24, AAL ID: 59); Precuneus_L (Voxels: 18, AAL ID: 67)
9	72	6	221	14	0.01	1.48	Thalamus_L (Voxels: 72, AAL ID: 77)
10	54	−25	266	7	0.032	1.20	Frontal_Inf_Orb_R (Voxels: 3, AAL ID: 16); Rectus_R (Voxels: 2, AAL ID: 28)
11	52	−10	250	71	0.043	1.00	Supp_Motor_Area_R (Voxels: 52, AAL ID: 20)
12	26	2	188	32	0.033	1.41	Precuneus_L (Voxels: 26, AAL ID: 67)
13	22	−7	239	70	0.046	0.78	Supp_Motor_Area_R (Voxels: 22, AAL ID: 20)
14	22	15	180	59	0.033	1.23	Parietal_Sup_L (Voxels: 19, AAL ID: 59)
15	18	−58	194	32	0.041	1.27	Angular_R (Voxels: 17, AAL ID: 66); SupraMarginal_R (Voxels: 1, AAL ID: 64)
16	13	−32	233	−3	0.046	1.10	Hippocampus_R (Voxels: 13, AAL ID: 38)
17	13	20	173	54	0.042	1.10	No AAL region matched
18	10	22	250	−2	0.043	1.24	Temporal_Pole_Sup_L (Voxels: 5, AAL ID: 83); Amygdala_L (Voxels: 1, AAL ID: 41)
19	10	5	195	6	0.04	1.36	Lingual_L (Voxels: 8, AAL ID: 47); Calcarine_L (Voxels: 2, AAL ID: 43)
20	8	−46	280	21	0.046	1.00	Frontal_Mid_R (Voxels: 6, AAL ID: 8); Frontal_Inf_Tri_R (Voxels: 2, AAL ID: 14)
21	6	−54	193	43	0.048	1.10	Parietal_Inf_R (Voxels: 6, AAL ID: 62)
22	4	−45	270	22	0.048	0.97	Frontal_Inf_Tri_R (Voxels: 4, AAL ID: 14)
23	4	−29	214	−1	0.048	1.09	ParaHippocampal_R (Voxels: 4, AAL ID: 40)
24	4	−28	217	0	0.045	1.23	Hippocampus_R (Voxels: 4, AAL ID: 38)
25	4	−31	219	−3	0.049	0.96	ParaHippocampal_R (Voxels: 4, AAL ID: 40)
26	3	−28	224	−2	0.049	0.93	Hippocampus_R (Voxels: 3, AAL ID: 38)
27	2	23	249	0	0.049	1.12	No AAL region matched
28	2	−14	196	6	0.048	1.04	Lingual_R (Voxels: 2, AAL ID: 48)
29	2	2	198	35	0.049	1.13	Cingulum_Post_L (Voxels: 1, AAL ID: 35); Precuneus_L (Voxels: 1, AAL ID: 67)
30	1	−34	237	−6	0.05	0.80	Hippocampus_R (Voxels: 1, AAL ID: 38)
31	1	−14	198	6	0.049	1.00	Lingual_R (Voxels: 1, AAL ID: 48)
32	1	−31	233	−5	0.049	0.92	Hippocampus_R (Voxels: 1, AAL ID: 38)
33	1	−12	198	6	0.049	0.98	Lingual_R (Voxels: 1, AAL ID: 48)
34	1	−47	271	23	0.049	0.90	Frontal_Inf_Tri_R (Voxels: 1, AAL ID: 14)
35	1	−31	213	−3	0.05	0.73	ParaHippocampal_R (Voxels: 1, AAL ID: 40)
36	1	−31	238	−5	0.048	1.21	Hippocampus_R (Voxels: 1, AAL ID: 38)

The GM regions that the cluster involves were identified according to the AAL3 atlas in the FSL software program. *p* values are shown after FWE correction. AAL3, anatomical automatic labeling 3; FSL, FMRIB Software Library; FWE, family-wise error; GM, gray matter; L, left; R, right.

Note that no significant clusters of NDI or ODI alterations were identified within the GP during the whole-brain GBSS analysis at the FWE-corrected threshold.

VOI-based regional analysis of the GP revealed no statistically significant differences between the HE and HC groups in terms of mean NDI (0.63 ± 0.10 vs. 0.69 ± 0.09, *p* > 0.05) or mean ODI (0.38 ± 0.07 vs. 0.38 ± 0.05, *p* > 0.05).

### 3.2 Significant group differences between the HE and HC group in terms of ODI

In patients with HE, a significantly increased ODI was found within the posterior cerebellum and cerebellar vermis (Table 3).

**Table 3:** Brain regions with significant differences in orientation dispersion index (ODI) between the hepatic encephalopathy (HE) and health control (HC) groups.

Cluster	Voxels	Coordinates			P_peak value_	Cohen’s d	AAL3 atlas
		X	Y	Z			
1	16	−2	208	−29	0.044	−1.28	Vermis_10 (Voxels: 7, AAL ID: 116); Cerebelum_9_R (Voxels: 6, AAL ID: 106); Vermis_9 (Voxels: 2, AAL ID: 115)
2	6	0	202	−33	0.045	−1.36	Cerebelum_9_L (Voxels: 5, AAL ID: 105); Vermis_9 (Voxels: 1, AAL ID: 115)

The GM regions that the cluster involves were identified according to the AAL3 atlas in the FSL software program. *p* values are shown after FWE correction. AAL3, anatomical automatic labeling 3; FSL, FMRIB Software Library; FWE, family-wise error; GM, gray matter; L, left; R, right.

### 3.3 Correlation between NODDI parameters of the GP and blood biochemical indices

**Figure 3:**
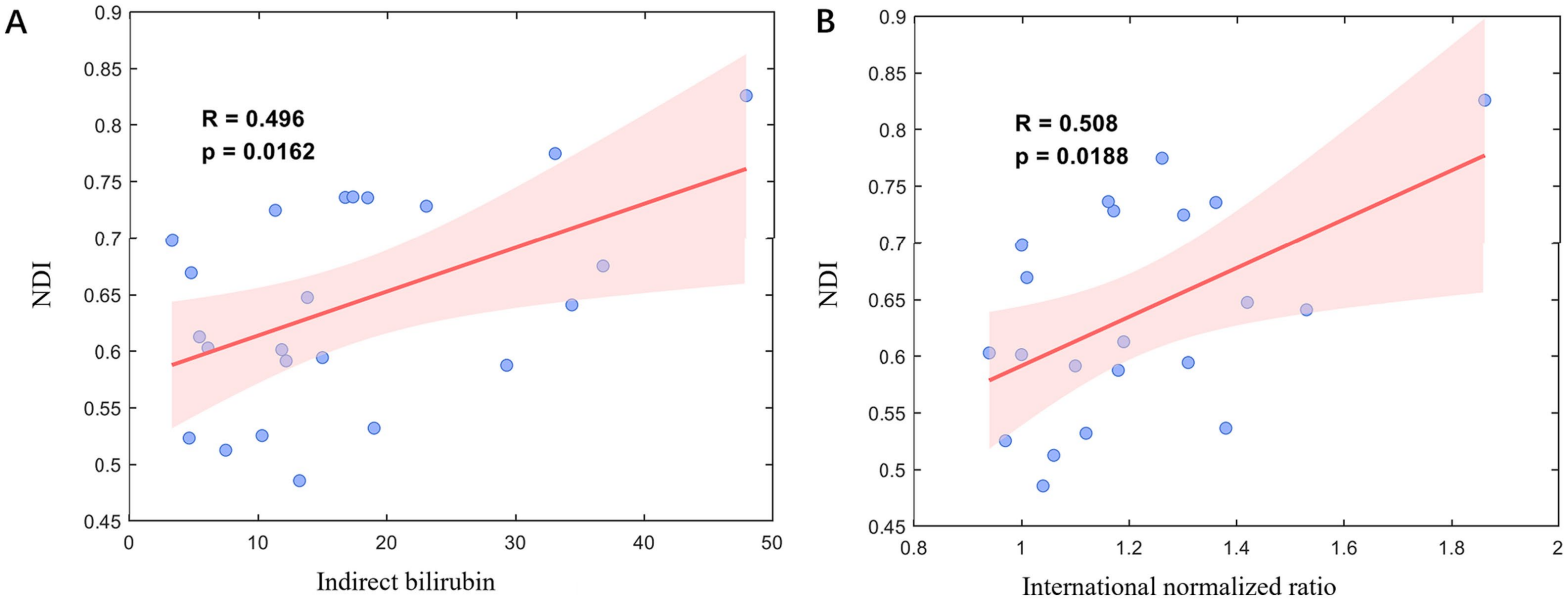
Correlations between right globus pallidus neurite density index (NDI) and **(A)** indirect bilirubin, **(B)** international normalized ratio (INR).

Pearson correlation analyses demonstrated that the NDI of the right GP was positively correlated with indirect bilirubin (*r* = 0.496, *p* = 0.016), and prothrombin international normalized ratio (INR) (*r* = 0.508, *p* = 0.019) (Figure 3).

**Figure 4:**
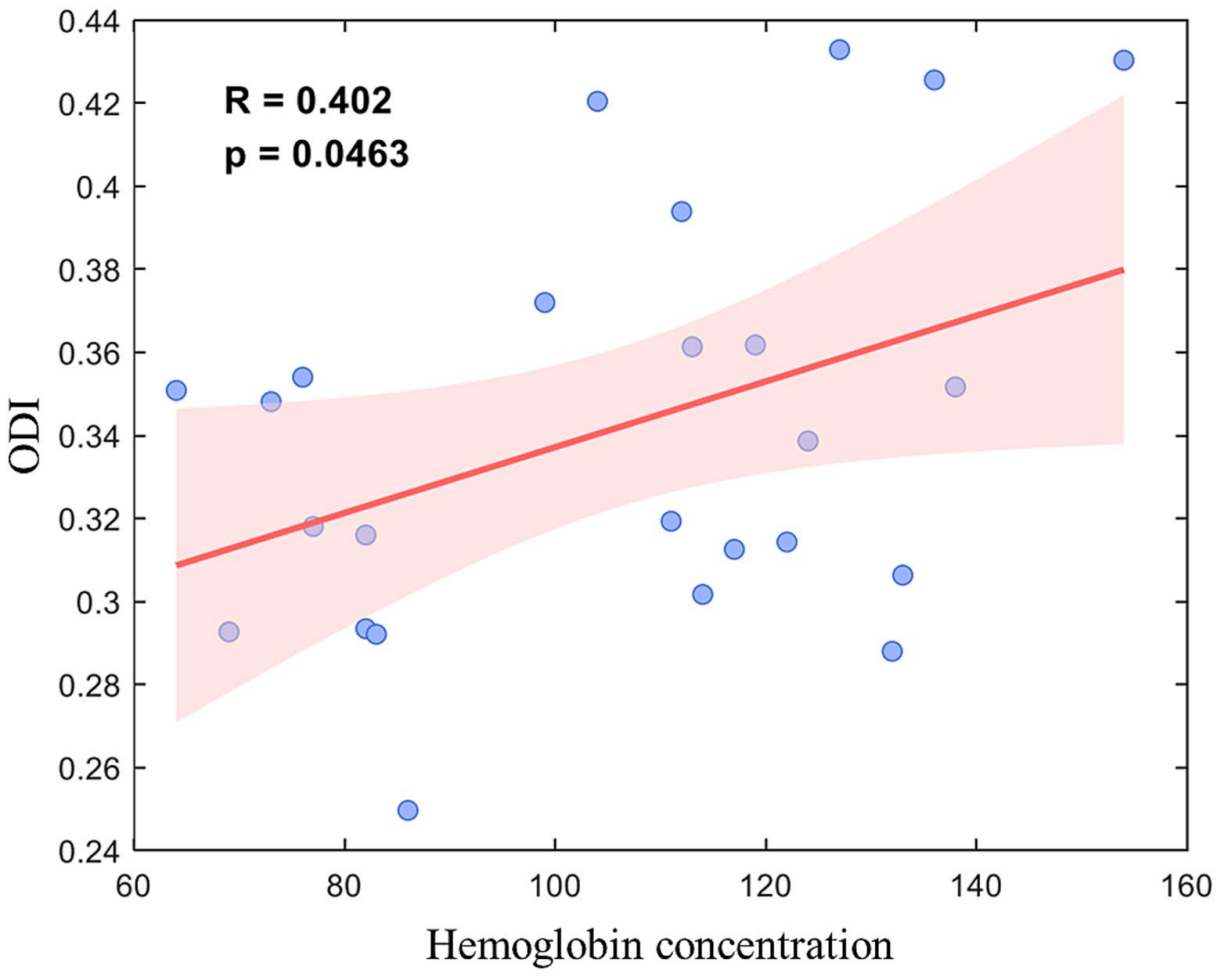
Correlations between left globus pallidus orientation dispersion index (ODI) and hemo-globin concentration.

Additionally, the ODI of the left GP was positively correlated with hemoglobin concentration (*r* = 0.402, *p* = 0.046) (Figure 4).

## 4 Discussion

In this study, we observed widespread neurite microstructural alterations in patients with HE, quantified using NODDI. HE patients showed significantly decreased NDI across multiple gray matter regions including frontal, temporal, parietal, occipital cortices as well as subcortical structures (thalamus, hippocampus, insula), and increased ODI primarily in cerebellar regions. Furthermore, correlations between GP and blood biochemical indices were also identified. These results provide novel biophysical insights into the neuropathology of HE, suggesting a complex interplay of neuroinflammation, edema, and metabolic toxicity.

The most prominent finding was the extensive reduction of NDI in the HE group, with the largest clusters located in the right medial superior frontal gyrus, right insula, and bilateral cingulate gyri. NDI serves as a sensitive marker for the intracellular volume fraction of axons and dendrites. The reduction of NDI in these regions likely reflects a combination of neurite atrophy and the expansion of the extracellular space due to low-grade cerebral edema (13). These regions, which play a central role in executive function, attention, and emotional regulation, may reflect the underlying neuronal damage and dysfunction in HE, consistent with cognitive decline observed in these patients (14, 15).

Interestingly, a recent GBSS-NODDI study (9) similarly identified reduced NDI patterns in patients with minimal hepatic encephalopathy (MHE), demonstrating significant NDI decreases specifically in the left insula and left middle frontal gyrus. These regions are involved in higher-order cognitive and multisensory integration. The consistency of these findings across both HE and MHE populations confirms that microstructural degeneration, detectable by NODDI, is a central feature of HE-related brain injury, beginning even at the clinically covert (MHE) stage. It is noteworthy that the spatial distribution of the most significant alterations differed between the studies, which may reflect the progression of neuropathology. In our HE group, the largest effect sizes were located in the right medial superior frontal gyrus, right insula, and bilateral cingulate gyrus. In contrast, the changes reported in MHE exhibited a left-hemisphere predominance. This pattern suggests a potential trajectory: early, subtle damage may preferentially affect or be more easily detected in left-hemispheric hubs of the executive and salience networks. As liver dysfunction progresses and clinical symptoms emerge, the damage becomes more bilateral and widespread, involving key midline structures, such as the cingulate cortex, and critical right hemisphere regions associated with attention and self-regulation, such as the medial prefrontal cortex.

The HE group also exhibited lower NDI in the precuneus compared to the HC group. The involvement of the medial prefrontal cortex and the precuneus highlights the vulnerability of the Default Mode Network (DMN). Consistent with our findings, previous resting-state functional MRI studies (16, 17) have confirmed the presence of dysfunction in the DMN in patients with HE, characterized by decreased amplitude of low-frequency fluctuation (ALFF) and attenuated functional connectivity. Disruption of the DMN is consistently associated with the cognitive deficits observed in HE, such as impaired attention and self-referential processing (18, 19). Our results extend these findings by pinpointing the biophysical basis of this dysfunction: a reduction in the model-derived neurite density index or integrity. Furthermore, the significant involvement of the insula (part of the Salience Network) supports the “network switching” hypothesis, where structural damage prevents the brain from effectively toggling between central executive and default mode states, leading to the behavioral rigidity seen in patients (20).

We observed a distinct pattern in the cerebellum, characterized by increased ODI in the posterior cerebellum and vermis. While reduced NDI indicates tissue loss, an increased ODI reflects a more disorganized or complex neurite configuration (9). In the context of HE, this increased complexity might represent a maladaptive response or “disorganized branching” in the face of ongoing neurotoxicity. The posterior cerebellum is increasingly recognized for its role in non-motor cognitive processes, including emotional regulation and attention (21, 22). The coexistence of reduced NDI and increased ODI suggests that the cerebellum undergoes active, albeit pathological, remodeling as part of a whole-brain network failure.

Bilirubin is a marker of liver failure severity; its correlation with GP NDI might reflect the synergistic effect of metabolic toxins on astrocyte swelling. An exploratory analysis of the GP was conducted due to its known vulnerability in HE. Although no significant group-level microstructural changes were detected in the GP, the correlation patterns suggest the dissociation between NDI and ODI alterations within the GP in relation to blood biochemical markers. This supports the notion that HE involves widespread pathophysiological mechanisms, including metabolic, inflammatory, and vascular derangements, which in turn affect neuronal microstructure (23). We observed that the NDI of the right GP was positively correlated with markers of liver dysfunction (indirect bilirubin and INR). Typically, neurodegeneration involves a reduction in neurite density. However, the GP is the preferential site for manganese (Mn) deposition and ammonium accumulation in patients with chronic liver disease, often manifesting as T1-weighted hyperintensity (24, 25). This accumulation often leads to astrocyte hypertrophy, which increases the apparent intracellular volume fraction (23, 26). Thus, the “pseudo-increase” in NDI likely reflects toxic cellular swelling rather than healthy neurite growth (27).

Conversely, the left GP ODI showed a positive correlation with hemoglobin concentration. Anemia is a common systemic complication of cirrhosis that exacerbates cerebral hypoxia (28). Our finding suggests that lower hemoglobin levels (more severe anemia) are associated with reduced ODI, representing a simplification of dendritic arborization or loss of synaptic complexity (29, 30). This allows us to propose a hypothesis a “double-hit” mechanism: toxic metabolic accumulation (bilirubin/manganese) drives cellular swelling and increases NDI, while systemic factors like anemia and hypoxia lead to a break-down of structural complexity (reduced ODI) (31) Care should be taken that these correlations should be viewed as exploratory associations; causal inference is not possible with the current cross-sectional design.

Furthermore, our findings should be interpreted within the broader context of metabolic alterations in HE. Previous MR spectroscopy (MRS) studies have consistently demonstrated a characteristic metabolic pattern in HE patients, marked by increased glutamine/glutamate (Glx) and decreased myo-inositol (mI) levels (32, 33). These shifts reflect osmotic abnormalities and astrocyte dysfunction: ammonia-induced glutamine accumulation in astrocytes leads to an osmotic gradient that draws water into the cells, resulting in low-grade brain edema (32, 34). These metabolic shifts provide a critical physiological backdrop for the microstructural changes detected by NODDI in our study, as osmotic stress and subsequent cellular swelling likely contribute to the increased NDI observed in the globus pallidus.

Several limitations in this study should be considered. First, the clinical heterogeneity of decompensated cirrhosis patients, including varying etiologies and liver dysfunction severity, may have introduced confounding factors. Future studies with larger, stratified cohorts are needed to enhance data homogeneity. Second, the sample size was relatively small for an exploratory neuroimaging analysis, which may limit the statistical power to detect subtle cortical alterations. Third, the cross-sectional design precludes the establishment of causal relationships between systemic biochemical changes and microstructural decay. Longitudinal research is essential to evaluate the efficacy of NODDI metrics in predicting disease progression. Fourth, blood ammonia levels were not available for the majority of HE patients due to incomplete clinical records, precluding correlation analyses between this key pathophysiological marker and NODDI metrics. Given the central role of hyperammonemia in HE pathogenesis, future studies should systematically collect ammonia data to clarify its relationship with microstructural alterations. Fifth, the 3.0 T MRI protocol may limit spatial resolution for smaller subcortical structures. Future studies using high-resolution, multi-shell diffusion protocols could further refine these findings. Despite these limitations, the present results provide meaningful insights into HE-related microstructural alterations.

## 5 Conclusion

This study employed the NODDI to characterize gray matter microstructural pathology in HE, revealing widespread microstructural alterations consistent with reduced neurite density index and cerebellar disorganization. The observed dissociated correlation patterns in the GP may be interpreted within the framework of a speculative “double-hit” hypothesis of neurotoxicity, although further longitudinal and mechanistic studies are required to validate this interpretation.

In conclusion, NODDI-derived parameters offer sensitive and biologically specific biomarkers that bridge systemic biochemistry and cerebral microstructure, holding promise for improving the early detection and mechanistic evaluation of HE.

## Data Availability

The raw data supporting the conclusions of this article will be made available by the authors, without undue reservation.

## Footnotes

1
https://www.nitrc.org/projects/mricrogl

2
https://github.com/arash-n/GBSS
